# Investigation the Effects of Green-Synthesized Copper Nanoparticles on the Performance of Activated Carbon-Chitosan-Alginate for the Removal of Cr(VI) from Aqueous Solution

**DOI:** 10.3390/molecules26092617

**Published:** 2021-04-29

**Authors:** Inas A. Ahmed, Hala S. Hussein, Ahmed H. Ragab, Najla AlMasoud, Ayman A. Ghfar

**Affiliations:** 1 Department of Chemistry, Faculty of Science, King Khalid University, Abha 62224, Saudi Arabia; ahrejab@kku.edu.sa; 2 Chemical Engineering & Pilot Plant Department, Engineering Division, National Research Centre, Cairo 11865, Egypt; hala.hussein21@yahoo.com; 3 Department of Chemistry, College of Science, Princess Nourah Bint Abdulrahman University, Riyadh 11671, Saudi Arabia; 4 Advanced Materials Research Chair, Chemistry Department, College of Science, King Saud University, Riyadh 11451, Saudi Arabia; Aghafr@ksu.edu.sa

**Keywords:** chromium, chitosan, green-zerovalent cupper, alginate, activated carbon, adsorption

## Abstract

In the present investigation, green nano-zerovalent copper (GnZVCu), activated carbon (AC), chitosan (CS) and alginate (ALG) nanocomposites were produced and used for the elimination of chromium (VI) from a polluted solution. The nanocomposites GnZVCu/AC-CS-alginate and AC-CS-alginate were prepared. Analysis and characterization were performed by the following techniques: X-ray diffraction, energy dispersive X-ray spectroscopy, scanning electron microscopy, transmission electron microscopy and Fourier transform infrared spectroscopy. The SEM analysis revealed that the nanocomposites are extremely mesoporous, which leads to the greatest adsorption of Cr^+6^ (i.e., 97.5% and 95%) for GnZVCu/AC-CS-alginate and AC-CS-alginate, respectively. The adsorption efficiency was enhanced by coupling GnZVCu with AC-CS-alginate with a contact time of 40 min. The maximum elimination of Cr^+6^ with the two nanocomposites was achieved at pH 2. The isotherm model, Freundlich adsorption isotherm and kinetics model and P.S.O.R kinetic models were discovered to be better suited to describe the exclusion of Cr^+6^ by the nanocomposites. The results suggested that the synthesized nanocomposites are promising for the segregation of Cr^+6^ from polluted solutions, specially the GnZVCu/AC-CS-alginate nanocomposite.

## 1. Introduction

The potential risk of heavy metal pollution to the environment has been arousing increasing public anxiety due to its extensive occurrence [1]. One of the most common contaminants is chromium, which is found in industrial wastewaters resulting from activities such as electroplating, metal finishing, leather tanning, textile activities and steel fabrication [2,3]. Moreover, it contaminates surface waters and groundwaters [4]. Naturally, Cr(VI) is present in two stable oxidation states, the first is Cr(VI) (hexavalent) and the second is a Cr(III) (trivalent) state. The first is much more toxic than the second [5,6]. Cr(VI) is considered harmful to humans when its concentration exceeds 0.05 mg·L^−1^ in drinking water [7]. Consequently, it is necessary to eliminate Cr(VI) anions from contaminated water [8]. Adsorption, chemical precipitation, biological reduction; ion exchange and adsorption are treatment processes that have been studied for Cr(VI) elimination [9,10,11].

Nowadays, using biomaterials as adsorbents is desirable due to their low cost and simple convenience [12]. For example, biopolymers are extensively applied in water-treatment owing to their wide variety of uses, biodegradability and ecological nature for instance [13]. Sodium alginate and chitosan are biopolymers with exclusive properties, for example hydrophilicity, biocompatibility, and non-toxic nature. Chitosan and alginate are polyelectrolyte polymers of opposite charges [14] and chosen as a source of biomaterial for the elimination of Cr^+6^.

Chitosan, a cationic, a hydrophilic polymer is obtained from chitin by deacetylation of aminoacetyl groups. Chitin is a major component of crustaceans’ shells, fungal cell walls and insects’ cuticle [15]. Alginate (ALG) is an anionic biopolymer that has receivedfurther consideration lately. It was studied as an adsorbent material for the elimination of water pollutants [16]. Because of itss properties such as hydrophilicity, biodegradability, and abundance in Nature, alginate is favored over other materials. Furthermore, the existence of carboxylate groups (–COOH) in alginate offers the capability to form complex multivalent ions. ALG has been modified into several forms with chitosan [17], cellulose, polyurethane, and activated carbon to improve its sorption capability for heavy metals removal from aqueous solutions and heavy metals have been successfully removed using a chitosan and sodium alginate composite [18].

Simultaneously, activated carbon has been used in environmental treatments because of the following properties: high surface area and functional groups [19]. For these reasons, activated carbon is ofterbcombined with composites for water treatment. Moreover, GnZVCu is added to activated carbon AC, CS and ALG to increase stability and efficiency. The green nZVCu is specifically chosen due to its catalytic activity and non-toxic nature [20]. The GnZVCu coupled with AC, CS and ALG were studied for the elimination of chromium Cr^+6^ from contaminated water.

The purposes of this study were to establish some new environmentally-friendly green nZVCu nanocomposites (i.e., GnZVCu/AC-CS-alginate and AC-CS-alginate). The analysis and description of the nanocomposite was performed using the SEM, XRD, EDX, TEM and FTIR techniques to investigate the adsorption capacity of the newly synthesized nanocomposites for hexavalent Cr^+6^. The chromium Cr^+6^ concentration was measured by a UV spectrophotometer. Different isotherm adsorption, kinetic models and thermodynamic parameters have been investigated to examine the adsorption process of chromium Cr^+6^ on the synthesized nanocomposites.

## 2. Results

### 2.1. Investigation of the Materials’ Properties

#### 2.1.1. Fourier Transform Infrared Spectroscopy Study

**Figure 1:**
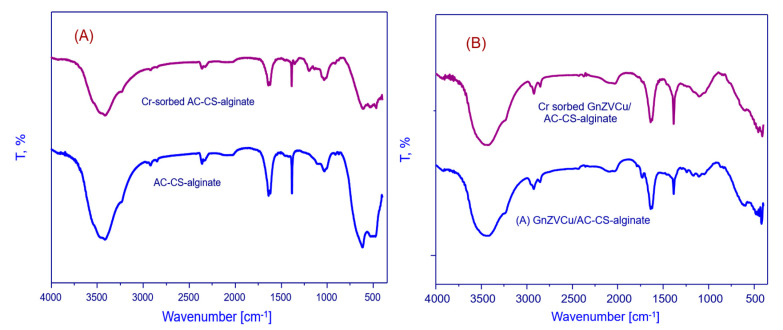
F.T.I.R analysis of (**A**) AC-CS-alginate, Cr-sorbed AC-CS-alginate, (**B**) GnZVCu/AC-CS-alginate, Cr sorbed- GnZVCu/AC-CS-alginate nanocomposite.

Figure 1 shows the Fourier transform infrared (FTIR) spectrum of the GnZVCu/AC-CS-alginate and AC-CS-alginate nanocomposites; before and after chromium Cr^+6^ adsorption. The broad peaks observed between 3200 and 3500 cm^−1^ were attributed to the intermolecular-hydrogen bonded O-H and N-H groups of chitosan, alginates and activated carbon in the nanocomposites [21]. The bands at 1619 and 1635 cm^−1^, are ascribed to C-C and C-O vibrations in the activated carbon and alginate, respectively. The expected peaks over 1620–1610 cm^−1^, ascribable to the amide groups of chitosan cannot be observed [22], because they are overlapped by the stronger COO- band of alginate [23]. The peaks observed at 1334 and 1031 cm^−1^ suggest the existence of OH bending and C-O stretching vibrations [24]. The non-appearance of the band related to the amine group (1160 cm^−1^) in the GnZVCu/AC-CS-alginate and AC-CS-alginate nanocomposites spectra validates the development of electrostatic-interactions between chitosan’s amino-groups (NH_3_^+^) with a positive charge and alginate’s carboxylic units (COO-) with a negative charge [25]. As demonstrated in Figure 1B, in Cr-sorbed GnZVCu/AC-CS-alginate, it was noticed that the -OH or NH_2_ stretching vibration bands at 3444 cm^−1^ moved to 3426 cm^−1^. This suggests the establishment of hydrogen bonds between the hydrogen atoms on the NH_2_ groups and O atoms of the oxyanionic Cr(VI) species [26]. In the meantime, the slight movement of the peak related to the (-COO) peak from 1635 to 1637 cm^−1^ is suggestive of interactions between (-COO) groups and Cr(VI). Meanwhile, Figure 1B recorded after Cr(VI) adsorption illustrates a slight shifting in the peaks seen at 1383 and 1031 cm^−1^ to 1384 and 1032 cm^−1^ indicative of interactions between the sorbent and Cr(VI). The previous dislocations describe the electrostatic-interaction between Cr(VI) and COO, OH, NH_3_^+^ groups. The wavenumbers (cm^−1^) for the dominant peaks from the FTIR study for Cr(VI) adsorption are listed in Table 1.

#### 2.1.2. X-ray Diffraction Study

**Figure 2:**
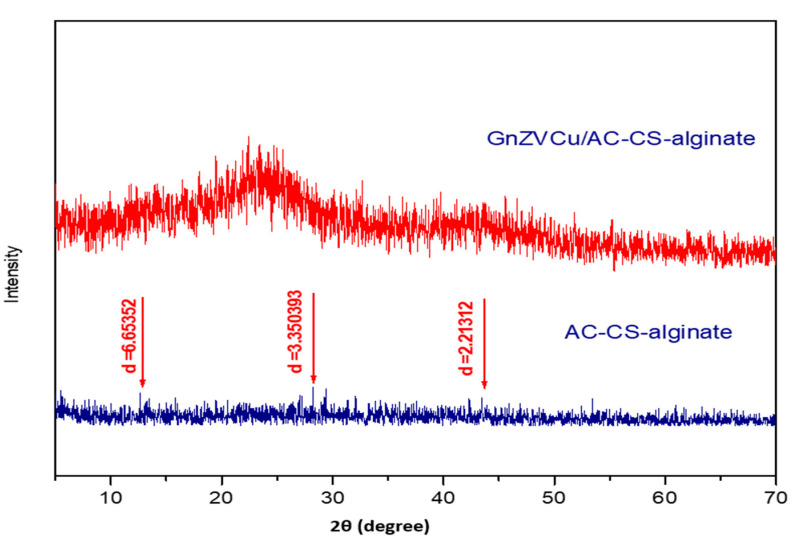
XRD pattern of AC-CS-alginate and GnZVCu/AC-CS-alginate nano-composite.

X-ray diffraction (XRD) analysis facilitates the determination of the crystalline or amorphous nature of the synthesized nanocomposites. The formation of the GnZVCu/AC-CS-alginate and AC-CS-alginate nanocomposites is displayed in Figure 2. The XRD analysis of the synthesized (GnZVCu/AC-CS-alginate shows a short broad peak obtained at 2θ = 23.5°. On comparing the XRD patterns, it was found that the hump appeared in GnZVCu/AC-CS-alginate XRD pattern indicates the composite is an amorphous material. The GnZVCu is a key factor in the accessibility to internal sites. Many studies have shown that decreasing the crystallinity causes an improvement in the heavy metal sorption properties [27]. From the observed results it is evident that the GnZVCu/AC-CS-alginate has a more amorphous nature which make it suitable for the adsorption process.

#### 2.1.3. Morphology and Elemental Composition of the Materials

**Figure 3:**
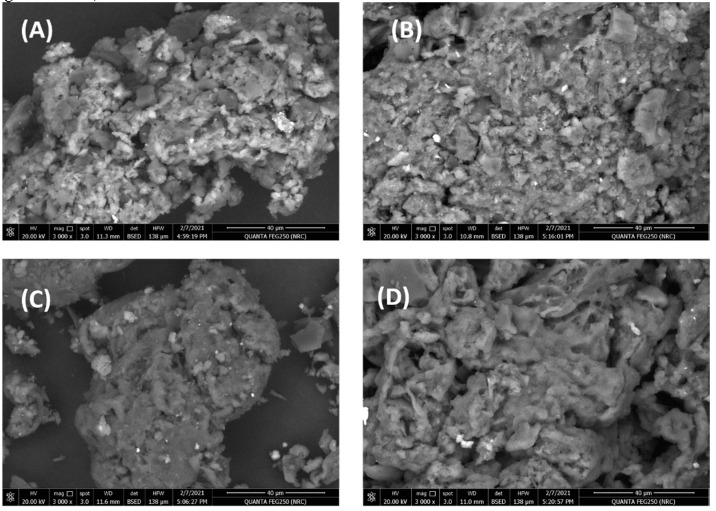
Shows SEM of (**A**) AC-CS-alginate, (**B**) Cr-sorbed AC-CS-alginate, (**C**) GnZVCu/AC-CS-alginate, (**D**) Cr-sorbed GnZVCu/AC-CS-alginate nano-composite.

The morphology and elemental composition of the synthesized GnZVCu/AC-CS-alginate and AC-CS-alginate nanocomposites before and after binding with Cr^+6^ were investigated. Figure 3 illustrates the surface morphology of all the synthesized nanocomposites, that were discovered to be rough and porous. The nanocomposite surfaces also displayed a significant number of widespread holes of various sizes. The surface properties, rough, porous and wide spaces are ascribed to the molecular diffusion [22]. Consequently, the synthesized porous nanocomposites will facilitate the high removal of Cr^+6^ from contaminated waters [28]. The surface morphologies of the (GnZVCu/AC-CS-Alginate) and (AC-CS-Alginate) nanocomposites were similar, as shown in Figure 3B. The Cu containing nanocomposite(GnZVCu/AC-CS-Alginate) shows nanostructures of Cu on its surface (Figure 3B,D).

**Figure 4:**
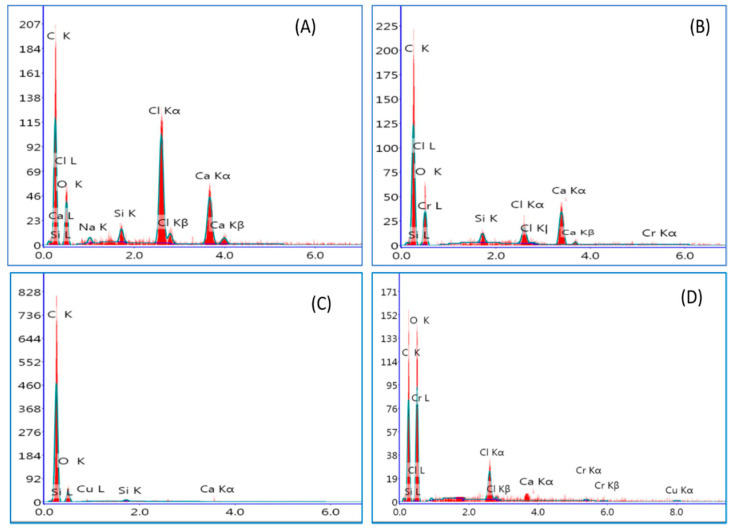
EDX of (**A**) AC-CS-alginate, (**B**) Cr-sorbed AC-CS-alginate, (**C**) GnZVCu/AC-CS-alginate, (**D**) Cr-sorbed GnZVCu/AC-CS-alginate nanocomposite.

Energy dispersive X-ray (EDX) evaluation confirmed the adsorption of Cr^+6^ ions on the nanocomposites’ surfaces. The EDX analysis of (GnZVCu/AC-CS-Alginate) and (AC-CS-Alginate) nanocomposite before and after binding with Cr^+6^ are shown in Figure 4. The EDX analysis of the (AC-CS-Alginate) and (GnZVCu/AC-CS-Alginate) nanocomposites before adsorption reveals peaks related to the elements carbon, oxygen and calcium (Figure 4A,C). Moreover, the EDX data verifies the existence of elemental Cu in the (GnZVCu/AC-CS-Alginate) nanocomposite. After adsorption, the EDX analysis proves the presence of chromium element in both composites. Thus, EDX verifies the trapping of Cr^+6^ ions on the (GnZVCu/AC-CS-Alginate) and (AC-CS-Alginate) nanocomposites surfaces via chelating and other linkages creating coordinated bonds. 

#### 2.1.4. TEM Analysis

**Figure 5:**
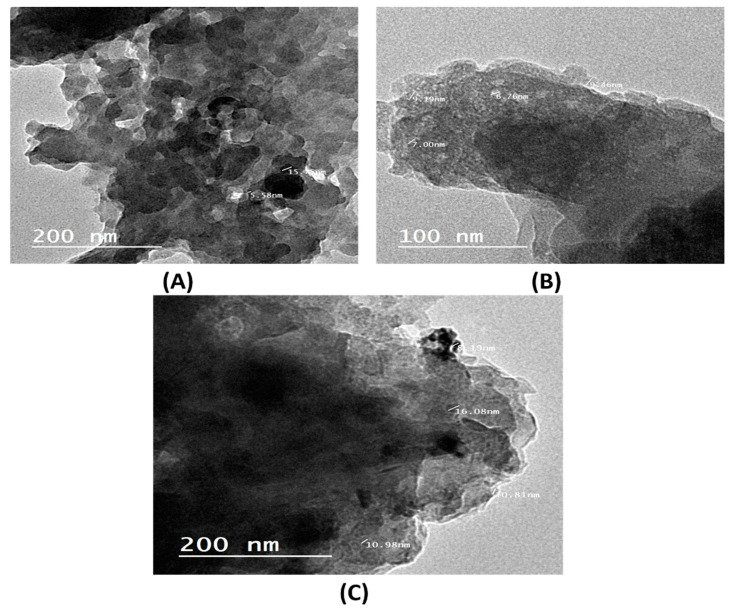
T.E.M. analysis of (**A**) (GnZVCu), (**B**) (AC-CS-Alginate), (**C**) (GnZVCu/AC-CS-Alginate) nanocomposite.

The TEM results of green nano-zerovalent copper (GnZVCu), (GnZVCu/AC-CS-Alginate) and (AC-CS-Alginate) nanocomposites are displayed in Figure 5. In the TEM image of green nano zerovalent copper (GnZVCu), it is obvious that the GnZVCu nanoparticles are reduced in size to a range from 5 to 15 nm and are homogenously dispersed with less accumulation. The TEM of (GnZVCu/AC-CS-Alginate) and (AC-CS-Alginate) nano-composites in Figure 5B,C display a dense and porous structure with a particle size of 8–16 and 6–10 nm, respectively. It is clear that when (GnZVCu) is added to (AC-CS-Alginate) nanocomposite, the mean particle size was slightly increased.

### 2.2. Performance of the Nano-Composites in the Removal of Cr^+6^

#### 2.2.1. Effect of pH on the Nanocomposite Performance

**Figure 6:**
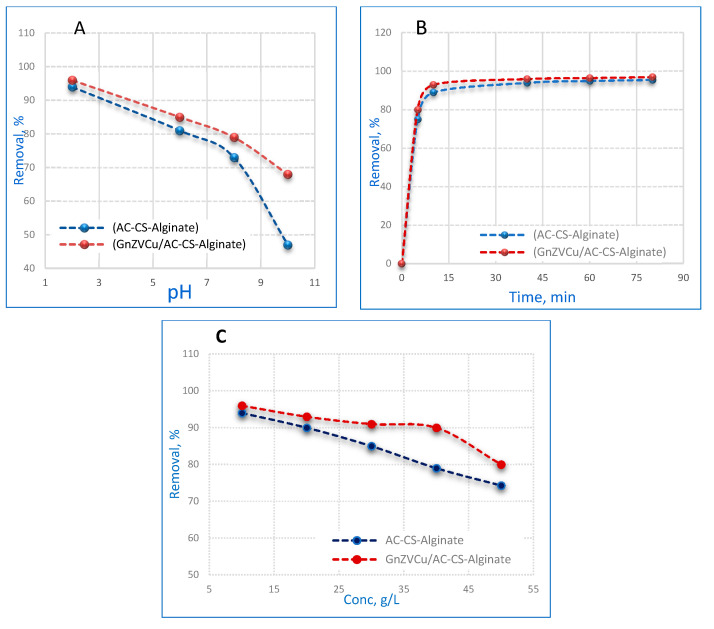
Effects of (**A**) (pH), (**B**) (contact time) and (**C**) (initial Cr(VI) concentration) on the adsorption of Cr (VI) by 0.4 g/50 mL nanocomposite.

The influence of pH on the (GnZVCu/AC-CS-Alginate) and (AC-CS-Alginate) nano-composites’ adsorption capacity was examined as the pH was varied from 2 to 8. The results in Figure 6A show that a high adsorption capacity of Cr^+6^ (96% and 94%) happens at low pH values for (GnZVCu/AC-CS-Alginate) and (AC-CS-Alginate) nanocomposites, respectively. Several investigators have recorded similar findings [29,30], whereby the maximum removal of Cr^+6^ occurred in an acidic medium at pH 2 and decreased after that. The explanation might be an increase in the electrostatic interaction between Cr^+6^ and the nanocomposites at pH 2. The most possible structures of Cr(VI) existing in solution are Cr_2_O_7_^2−^, CrO_4_^2−^, HCrO_4_^−^ and H_2_CrO_4_, which depend on the solution pH, the concentration of Cr(VI) and redox potential [31]. In acidic solution; where the pH extends from 2 to 4, HCrO_4_^−^ is the most important Cr(VI) species [32], while in a neutral medium with a pH varying from 4 to 7, CrO_4_^2−^ is the main phase. The beneficial influence of low pH can be ascribed to the negative charge neutralization on the nanocomposite surface by additional hydrogen ions, thus accelerating the diffusion of hydrogen chromate ions (HCrO_4_^−^) and their consequent adsorption. The lower removal efficiency of Cr^+6^ by both nanocomposites at pH > 2 might be ascribed to better movement of H_3_O^+^ in the solution that subsequently competes against Cr^+6^. The adsorption capacity of (GnZVCu/AC-CS-Alginate) is higher compared with (AC-CS-Alginate) nanocomposites, which is related to the fact that zerovalent Cu provides electrons to the solution, which reduces the donation of protons in the solution and accordingly, causes an increase in the electrostatic interaction between the Cr^+6^ and the nanocomposites [33].

#### 2.2.2. Contact Time Effect

The contact time effect on Cr^6+^ adsorption by both (GnZVCu/AC-CS-Alginate and (AC-CS-Alginate) nanocomposites is presented in Figure 6B. The elimination percentage of Cr^6+^ ions was measured at specific time points varying between 5 to 80 min with an initial concentration of 10 ppm Cr^6+^ and pH 2 utilizing 0.4 g/50 mL of nanocomposite. Figure 6B shows that the adsorption of chromium was fast for the first 10 min and then it continued at a slower rate until it reached saturation. The early fast rate might be related to the large number of vacant sites present during the early phase and in time the surface sites become exhausted. Within 10 min, the percentage removal of Cr^6+^ was up to 90%. The equilibrium was established after 40 min. One may draw a conclusion based on these findings that (GnZVCu/AC-CS-Alginate and (AC-CS-Alginate) nanocomposites are effective adsorbents of Cr^6+^ from an aqueous solution.

### 2.3. Effect of Cr (VI) Ion Concentration

The impact of the Cr^6+^ concentration on the adsorption process was investigated from 10 to 50 ppm with 0.4 g/50 mL of nanocomposite, 40 min contact time and a pH = 2, as characterized in Figure 6C. It was found that Cr(VI) adsorption increased when the chromium concentration decreased for both that (GnZVCu/AC-CS-Alginate and (AC-CS-Alginate) nanocomposites. This behavior is attributed to the fact that there are more active sites in the nanocomposites adsorbents’ surfaces available for chromium retention at high dilution. It is seen that as the initial concentration of Cr^6+^ increased, the absorption capacity of AC-CS-Alginate was lowered in contrast with that of the GnZVCu/AC-CS-Alginate nanocomposite. This is may be related to the presence of zerovalent copper in (GnZVCu/AC-CS-Alginate) nanocomposite that donates electrons to the aqueous solution, which tends to improve the absorptivity of Cr^6+^ ions.

### 2.4. Kinetic Models

The kinetics data for the adsorption of Cr(VI) on (GnZVCu/AC-CS-Alginate) and (AC-CS-Alginate) nanocomposites were examined via three several kinetic models [34] (pseudo-first order reaction (PFOR), pseudo-second order reaction (PSOR) and intraparticle diffusion models). The best conditions were fixed as pH 2, the amount of nanocomposites were 0.4 g/50 mL, the contact time was 40 min, and 10 ppm was selected as the initial Cr^6+^ concentration.

#### 2.4.1. Pseudo-First Order Reaction Kinetics

**Figure 7:**
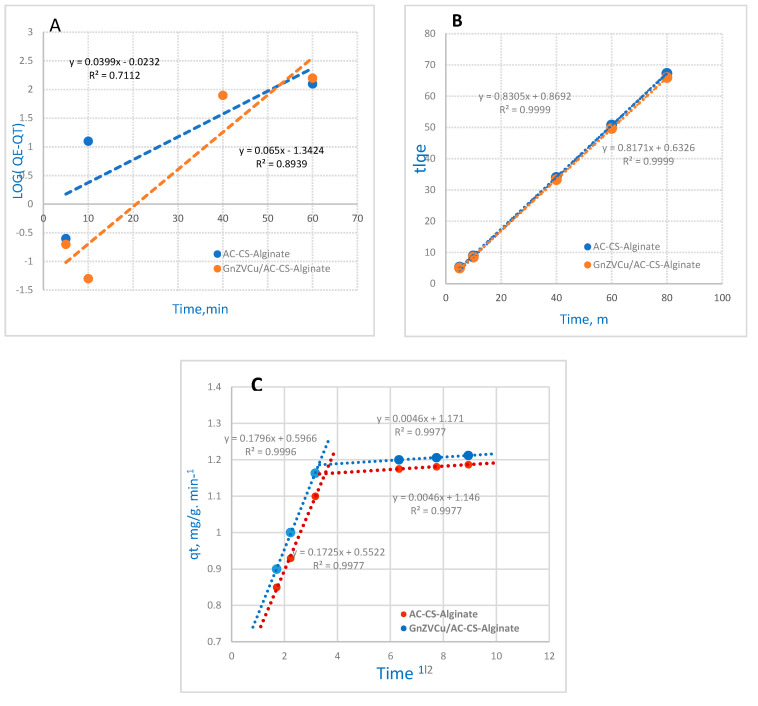
The adsorption kinetics: (**A**) (P.F.O.R.), (**B**) (P.S.O.R.), (**C**) Mories−Weber equation for Cr(VI) adsorption on GnZVCu/AC−CS−Alginate and AC−CS−Alginate nano-composites (sorption time 40 min; sorbent dosage 0.4 g/50 m L, pH = 2).

The reaction kinetics related to a PFOR are presented in Figure 7 and can be calculated by the following equation [35]:

Log (q_e_ − q_t_) − log q_e_ = − K_ads_ t/2.303 (1)

where the adsorption capacity at time t is denoted by (q_t_ in mg/g) and (k_ads_ min^−1^) is the rate constant of PFOR adsorption.

In this work, the uptake of Cr^6+^ on GnZVCu/AC-CS-Alginate) and (AC-CS-Alginate) nanocomposites displayed a linear relationship. The slope and intercept data were obtained by plotting log (q_e_ − q_t_) against the contact time (t) and hence calculating the values of (q_e_) and (k_ads_). Figure 7A shows the PFOR kinetics. Moreover, Table 1 illustrates the PFOR correlation coefficients (R^2^) which are low for both (GnZVCu/AC-CS-Alginate and (AC-CS-Alginate) nanocomposites. In addition, there is a wide gap between the theoretical and experimental value of the equilibrium adsorption (q_e_), so the application of the PFOR model is deemed unsuitable.

#### 2.4.2. Pseudo-Second-Order Reaction Kinetics

The PSOR kinetics model is calculated by the following equation [36]:

t/q = 1/K_2_q_e2_ + t/q_e_. (2)

where k_2_ (g/mg/min) is the rate constant of the reaction. Figure 7B illustrates the relation between t/q_t_ against t, the rate constant (k_2_) and equilibrium adsorption capacity (q_e_) represented by slopes and intercept values. Additionally, R^2^ values (the correlation coefficient) can be obtained. The obtained data display high a R^2^ (0.9999) for the (GnZVCu/AC-CS-Alginate) and (AC-CS-Alginate) nanocomposites. The collected data are summarized in Table 1. Also, the q_e_ value, that was calculated, is compatible with the experimental data of the PSOR kinetics for both composites. Consequently, the adsorption for the (GnZVCu/AC-CS-Alginate) and (AC-CS-Alginate) nanocomposites fit well to pseudo-second order kinetics.

#### 2.4.3. Mories–Weber Equation

Mories–Weber equation illustrates the intraparticle-mass-transfer-diffusion model [37] (see Figure 7C):

q = Kd (t)1/2.(3)

where q (g/g) signifies the adsorbed Cr^+6^ ions, K_d_ means the intraparticle-mass-transfer-diffusion-rate constant, and t1/2 corresponds to the square root of time. Then, if the results are compatible with the intraparticle-diffusion, it is the only desirable step. Figure 7C illustrates the Morris-Weber kinetic equation plot. From the obtained data the first part of the relation is linear, that perhaps refers to the effect of the boundary layer. However, the second part is probably related to the effect of intraparticle diffusion [38]. These data indicate that all the uptake processes occur within the first 40 min in a certain linear way. Hence, the results confirm that the porosity of the nanocomposites superceded the effects of resistance to intraparticle diffusion [39]. From the calculations, the intraparticle diffusion rate constant (K_d_) was 0.1725 and 0.1796 (g/g·min^–1^)) for the (AC-CS-Alginate) nanocomposite and (GnZVCu/AC-CS-Alginate) nanocomposite, respectively, revealing the interaction of Cr (VI) with both composites. From these findings, it is clear that the kd value of (GnZVCu/AC-CS-Alginate) nanocomposite is higher compared with that of (AC-CS-Alginate) nanocomposite. The data of the PFOR, PSOR, and Mories–Weber kinetic model calculations are presented in Table 2.

### 2.5. Isotherm Model

Isotherm model are required to interpret the adsorption process adequately [40]. Among the isotherm models the Freundlich, Dubinin-Radusekevisch-Kanager and Langmuir models were utilized to test the results. The optimum experimental conditions were selected as pH 2, the amount of GnZVCu/AC-CS-Alginate and AC-CS-Alginate nano-composites was 0.4 g/50 mL and the initial concentration of Cr(VI) was 10 ppm with a contact time of 40 min. The Langmuir isotherm is applied to elucidate substance adsorption via a homogenous surface with nominal interface among the adsorbed molecules [41]. This model assumes a regular adsorption on the surface of nanocomposites with a high value, based on the saturation level of the monolayer. The Langmuir model is characterized by the following linear equation [42]:

Ce/q_e_ = 1/K_L_.q_max_ + (1/q_max_).C_e._ (4)

**Figure 8:**
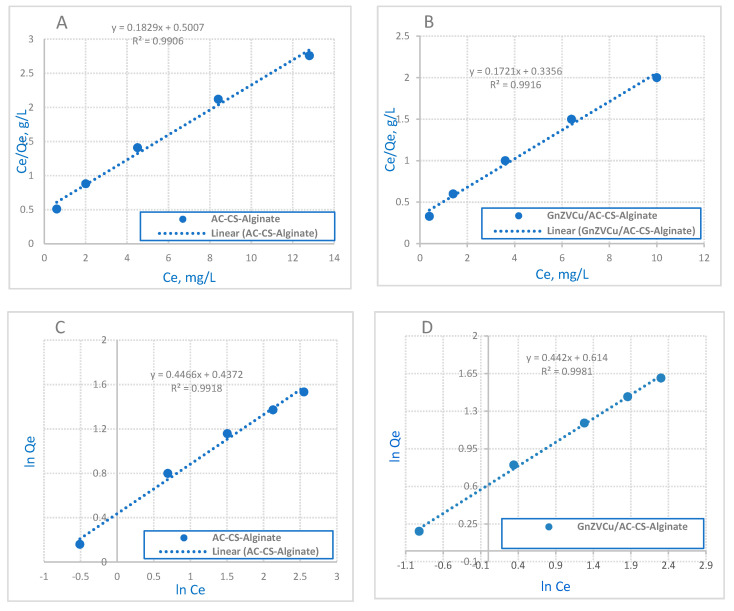
The Langmuir adsorption for Cr ion removal on (**A**) AC-CS-Alginate nanocomposite and (**B**) GnZVCu/AC−CS−Alginate nanocomposite and Freundlich adsorption for Cr ion removal on (**C**) AC−CS−Alginate nanocomposite and (**D**) GnZVCu/AC−CS−Alginate nanocomposite (sorption time: 40 min; sorbent dosage: 0.4 g/50 mL, pH = 2).

K_L_ (L·mg^−1^) identifies the capacity for monolayer adsorption of the heat of sorption, and the maximum adsorption capacity (q_max_ (mg·g^−1^)). Figure 8A,B illustrate the Langmuir adsorption isotherm, which is dependent on the monolayer adsorption of the adsorbate on the surface of adsorbent throughout the adsorption process. The Langmuir adsorption isotherm generally determines the equilibrium uptake of the homogeneous surface of adsorbents.

The Freundlich model is a primary empirical equation that is convenient to establish the exponential distribution of active centers. Moreover, it is exact for heterogeneous surfaces [43,44]; the model equation is represented as shown below:

ln q_e_ = ln K_f_ + 1/n ln C_e_. (5)

Here K_f_ is identified the adsorption capacity and n is a measure of the intensity. Moreover, K_f_ is a proportional value for the adsorption capacity. Also, (n) means a favorable adsorption extent, so if the (n) value is higher than 1, it will assure the compatible nature of adsorption [45]. The data reveal that the Freundlich model is a better fit than the Langmuir model for both nanocomposites. The correlation coefficient (R^2^) values are listed in Table 2. Additionally, the value of R^2^ of a Freundlich model plotg for both (GnZVCu/AC-CS-Alginate) and (AC-CS-Alginate) is 0.9981 and 0.9918, respectively, which is higher than that obtained by a Langmuir isotherm. Also, the adsorption capacities suggest that the uptake of Cr ions may involve multilayer coverage on the (AC-CS-Alginate) and (GnZVCu/AC-CS-Alginate) surfaces (1.54 and 1.84 mg/g). Hence, the obtained data are a good match with the Freundlich model.

### 2.6. The (D-R) Isotherm

The Dubinin–Radusekevisch–Kanager isotherm is suitable for both adsorption processes and Gaussian energy distribution calculations on a heterogeneous surface. The model equation (D-R) is represented as the following expression [46]:

ln q = ln q_(D-R)_ − βε^2^ (6)

ε = RT ln(1+1/C_e_) (7)

where: q_(D-R)_ (mg·g^−1^) corresponds to the theoretical adsorption capacity, whereas the β is equivalent to the activity coefficient (mol^2^ kJ^−2^) the mean sorption energy, ε is the Polanyi potential), R is the ideal gas constant 0.008314 kJmol^−1^K^−1^ and T is the absolute temperature in K:

E = 1/(2β)^1/2^. (8)

E (kJ mol^−1^) is known as the free energy change. The E value could be calculated to know the kind of reaction. Hence, If E < 8 kJmol^−1^, it will be expected that the physical forces may affect the adsorption process, while, if E lies between 8 to 16 kJmol^−1^, this means that the sorption process is governed by chemical ion exchange. Also, if E > 16 kJmol^−1^, it is predicted that the sorption is under particle diffusion control [47]. All results of the D-R models are summarized in Table 3. The E values are 0.7593 and 0.7383 kJ mol^−1^ for Cr^+6^ ions absorption onto the (AC-CS-Alginate) and (GnZVCu/AC-CS-Alginate) nanocomposites, respectively, so since E < 8 kJmol^−1^, the data suggest that the sorption process proceeds via physical adsorption [48].

### 2.7. Sorption Thermodynamics

The thermodynamic parameters standard-free energy (∆G^o^), standard- enthalpy (∆H^o^) and standard-entropy (∆S^o^) were applied to evaluate the thermodynamic feasibility and spontaneous nature of the adsorption process. These items were studied to explain the thermodynamic action of the removal of Cr^+6^ ions that are adsorbed on (AC-CS-Alginate) and (GnZVCu/AC-CS-Alginate) nanocomposites. The obtained outcomes were recorded at various temperatures (27, 40, and 50 °C). Then, the thermodynamic items were calculated via the following equation [48,49]:

∆G^o^ = –RT ln K_d_ (9)

∆G^o^ = ∆H^o^ − T∆S^o^ (10)

LnK_d_ = −∆H^o^/RT + ∆S^o^/R (11)

where R is the gas constant is (8.314 Jmol^−1^K^−1^), T the absolute temperature (K) and K_d_ the distribution coefficient. The data of ∆G^o^ was calculated by Equation (9). Also, the thermodynamic items ∆S^o^ and ∆H^o^ were computed by Equation (10). From these results, it can be concluded that the amount of Cr^+6^ ion uptake by both nanocomposites decreased as the temperature was increased. This may be explained by the fact the increase in temperature will alter the contaminants’ solubility in a bulk solution to a larger extent than contrarily the adsorption of Cr^+6^ ions [50]. The values of the thermodynamic parameters for the sorption of Cr^+6^ ions on the (AC-CS-Alginate) and (GnZVCu/AC-CS-Alginate) nanocomposites are displayed in Table 4.

The negative values of ∆G^o^ assure that the adsorption process was feasible and spontaneous. Furthermore, the negative values of ∆H^o^ show that the Cr^+6^ adsorption onto (AC-CS-Alginate) and (GnZVCu/AC-CS-Alginate) nanocomposites was exothermic in nature. The negative values of ∆S^o^ for (AC-CS-Alginate) and (GnZVCu/AC-CS-Alginate) nano-composites shows that the randomness decreased at the solid–liquid interfaces as a result of Cr^+^ adsorption onto the adsorbents’ surfaces. This implied that the adsorption process was energetically stable [51]. At high temperatures, the nZVCu composite was shown to be very stable compared with (AC-CS-Alginate) nanocomposite. The sorption tends to be physical-sorption as indicated by the ΔG^o^ values less than 80 kJ mol^−1^. However, it may be chemisorption when ΔG^o^ ranges from 80 to 400 kJ mol^−1^ [52], so from the ΔG^o^ values that are listed in Table 5, the sorption of the (AC-CS-Alginate) and (GnZVCu/AC-CS-Alginate) nanocomposites is presumed ti be due to physical sorption. These results coincidence with the D-R isotherm results.

## 3. Materials and Methods 

### 3.1. Materials

The materials were carried out in this research contained extra pure activated carbon, chitosan purchased from (Al-Gomhoria Company, Al-Mansoura City, Egypt), Sodium alginate, copper (II) sulphate pentahydrate (CuSO_4_·5H_2_O), potassium dichromate(K_2_Cr_2_O_7_) and calcium chloride (CaCl_2_) were obtained from Sigma-Aldrich (Cairo, Egypt). All the substances were of commercial grade and used without any purification.

#### 3.1.1. Synthesis of Copper Nanoparticles

The production of green copper nanoparticles (GnZVCu) was performed using green tea leave extracts, according to the technique explained by Asghar [62]. Green tea leaves were purchased from a local market in Cairo (Egypt). Copper nanoparticles from green tea were produced utilizing CuSO_4_ with tea leaves extracts. Briefly, CuSO_4_ (1 mmol/L) and tea leave extracts were used in a 4:1 ratio by volume, and the solution was subjected to continuous stirring at 80 °C for 10 min. The resultant suspensions were settled at room temperature for 24 h to complete the reaction, then filtered and washed three times with DI-H_2_O to remove any unbound molecules. Lastly, Cu-NPs were dried at 65 °C for 3 h.

#### 3.1.2. Preparation of GnZVCu/AC-CS-Alginate Nanocomposites

**Figure 9:**
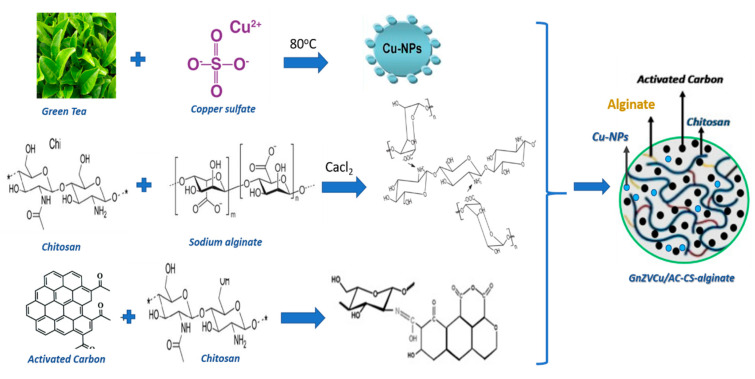
The synthesis mechanism of copper nanoparticles-modified AC-CS-alginate composites.

Sodium alginate (ALG) and calcium chloride (CaCl_2_) were dissolved in distilled water. The pH of the sodium alginate solution was adjusted to 5.1 using hydrochloric acid. In brief, 2 g of chitosan (CS) was mixed with 1% (acetic acid solution). The nanocomposite was created by mixing in a beaker with stirring GnZVCu (1.0 g), activated carbon (2.5 g), CS (2 g), and ALG (2.5 g) in the order of CS solution followed by ALG solution, and subsequently activated carbon (AC) and finally GnZVCu. A solution of calcium chloride (2 mL, 3.35 mg/mL) was then added dropwise to the beaker with continuous stirring for 2 h at room temperature using a disposable syringe. Afterwards, the obtained GnZVCu/AC-CS-alginate nanocomposite was left in CaCl_2_ solution overnight with mild stirring, then washed various times and dried at 40 °C for one day. The other composite; AC-CS-alginate was prepared using the same procedure described above. Desiccators were used to store all of the nanocomposites that had been prepared [62]. The synthesis mechanism of copper nanoparticles-modified AC-CS-alginate composites is shown in Figure 9.

### 3.2. The Method of Chromium (VI) Analysis

The dissolved hexavalent chromium can be determined calorimetrically by reaction with diphenylcarbazide in acid solution (PH 1–3). The reaction was conducted by mixing of 2 mL diphenylcarbazide and 100 mL of diluted hexavalent chromium solution, and allowing the mixture to stand for 5–10 min. until a red-violet color was formed. The reaction is very sensitive, and the absorbance index per gram atom of chromium was measured at 540 nm.

### 3.3. The Nanocomposites Surface Characterization

#### 3.3.1. Instruments

FTIR-spectroscopy (Genesis-II FT-IR spectrometer, ALT, San Diego, CA, USA) was used to illustrate the synthesized nano-composites. Moreover, scanning electron microscopy (SEM, Inspect S, FEI Company, Eindhoven, the Netherlands) and EDX (Quanta 200, FEL) tests were performed. Transmission electron microscopy (TEM) utilizing a JEM-HR-2001 model instrument (JEOL, Akishima, Japan) with an accelerating voltage of 200 kV was applied to gauge the materials’ particle sizes. In order to determine the materials’ compositions, X-ray diffraction (XRD) using a Philips PW 1050/70 diffractometer (Philips, Amsterdam, the Netherlands) was used.

#### 3.3.2. Adsorption Examinations of Hexavalent Chromium Cr^+6^

In this study potassium dichromate (K_2_Cr_2_O_7_) was used as the source of Cr^+6^ ions. The Cr^+6^ adsorption method was as follows: 50 mL of Cr^+6^ solution was mixed with nanocomposite under various conditions. The experimental factors such as pH, temperature, time, and Cr^+6^ concentration adsorption performance on GnZVCu/AC-CS-alginate and AC-CS-alginate nanocomposites were examined. The value of the chromium Cr^+6^ pH was modified by applying dil. HCl and NaOH. The investigational experiments were conducted at (27 ± 1 °C). The variables examined were the solution pH (2, 6, 8, and 10), chromium Cr^+6^ initial concentration (10, 20, 30, 40, and 50 ppm), time of contact (5, 10, 40, 60, and 80 min) and amount of sorbents (0.4 g/50 mL). The quantity of adsorbed metal (q_e_) was defined by following Equations [63].

(12) $$\mathrm{Adsorption}\;\mathrm{Capacity}\;q_{e}\;=\;\frac{\left(C_{o\;}-C_{e}\right)V}{W}$$

(13) $$\mathrm{Cr}\;\mathrm{removal}\;\mathrm{efficiency}\%\;=\;\frac{\left(C_{o\;}-C_{e}\right)}{C_{o\;}}\;\times \;100$$

where *_Co_* and *_Ce_* are the initial and equilibrium concentrations (mg/L) of *Cr* (VI) ions; q_e_ (mg/g) implies the equilibrium adsorption capacity; W (g) and V (L); are adsorbent weight and solution volume, respectively.

## 4. Conclusions

In the present study, (AC-CS-Alginate) and (GnZVCu/AC-CS-Alginate) were effectively prepared and used for the elimination of Cr^+6^ from contaminated solutions and analyzed through FTIR, SEM, XRD and TEM. The GnZVCu composite showed high performance in the removal of Cr^+6^, and was also found to have better stability and loading efficiency at high temperature compared with(AC-CS-Alginate) nanocomposite. The coupling of GnZVCu with AC-CS-Alginate was noticed to decrease the crystallinity, and thus lead to the highest elimination of Cr^+6^ as compared to (AC-CS-Alginate). The removal capacity is mostly reliant on the initial concentration of the heavy metal and the solution pH. The optimum pH for the elimination of Cr^+6^ from aqueous solution was pH 2. The adsorption and kinetic models of Cr^+6^ adsorption onto (AC-CS-Alginate) and (GnZVCu/AC-CS-Alginate) nanocomposites was discovered to fit a Freundlich adsorption and pseudo second-order rate equation well. The reaction was found to be exothermic and spontaneous. The sorption mechanism of (AC-CS-Alginate) and (GnZVCu/AC-CS-Alginate) nanocomposites is physical sorption. The developed composites displayed the ability to be useful as adsorbent materials for water treatment.

## Figures and Tables

**Table 1:** Wavenumbers (cm^−1^) for the dominant peaks from FTIR study for Cr(VI) adsorption.

S. No	Frequency (cm^−1^)	Functional Group
1	3426 cm^−1^	OH or NH_2_ stretching
2	1637 cm^−1^	COO stretching
3	1619 cm^−1^	C-C stretching
4	1334 cm^−1^	O-H bending
5	1031 cm^−1^	C-O stretching

**Table 2:** Calculations of P.F.O.R., P.S.O.R., and Mories–Weber equations.

Kinetic Models	Parameter	(AC-CS-Alginate)	(GnZVCu/AC-CS-Alginate)
PFOR	q_e_, exp (mg g^−1^)	1.187	1.212
	q_e_, cal (mg g^−1^)	0.9479	0.0454
	K_ads_ (min^−1^)	1.0962	1.1614
	R^2^	0.7112	0.8939
PSOR	q_e_, cal (mg g^−1^)	1.2041	1.2233
	K_2_ (g mg^−1^ min^−1^)	0.9555	1.2925
	R^2^	0.9999	0.9999
Mories–Weber	K_d_ (mg g^−1^ min^0.5^)	0.1725	0.1796
	R^2^	0.9977	0.9996

**Table 3:** Kinetic Isotherm.

Kinetic Isotherm	Parameter	(AC-CS-Alginate)	(GnZVCu/AC-CS-Alginate)
Langmuir	q_e_, exp (mg g^−1^)	1.187	1.212
	q_e_, cal (mg g^−1^)	5.467	5.810
	K_L_ (L mg^−1^)	0.365	0.513
	R^2^	0.990	0.991
Freundlich	K_F_ (mol^n−1^ L^n^ g^−1^)	1.54	1.84
	N	2.24	2.260
	R^2^	0.9918	0.9981
D-R model	E (kJ mol^−1^)	0.7593	0.7383
	q(D-R) (mg g^−1^)	2.705	2.768
	R^2^	0.9932	0.9983

**Table 4:** The data of thermal parameters of Cr^+6^ adsorption (10 ppm of 0.4 g/50 mL of nanocomposites, for a contact time of 40 min and at a pH of 2).

Parameter	T (K)	A%	LnK_L_	∆H^o^ (KJ.mol^−1^)	∆S^o^ (J.mol^−1^.K^−1^)	∆G^o^ (kJ.mol^−1^)	R^2^
**Cr^+6^/(AC-CS-Alginate)**	300	94	0.672	−23.393	−70.729	−1.619	0.9917
	313	90.5	0.174			−0.455	
	323	87	−0.178			0.481	
**Cr^+6^/GnZVCu/AC-CS-Alginate**	300	96	1.098	−21.461	−61.633	−2.747	0.9993
	313	94	0.672			−1.754	
	323	91.7	0.322			−0.867	

**Table 5:** Adsorption performance of Cr (VI) onto different adsorbents.

Metal	Adsorbent	q_e_ (mg/g)	Reference
Cr (VI)	Modified activated carbon	18.51	[53]
	Graphene oxide–manganese ferrite (GMF) nanomaterials	34.02	[54]
	Chitosan-based hydrogel	93.03	[55]
	Synthesize dMgO/Fe_3_O_4_ nanocomposite	23.90	[56]
	Activated carbon from *Leucaena leucocepnala*	13.85	[57]
	Fe_3_O_4_-NH_2_ (amino functionalized magnetic nano-adsorbent	232.51	[58]
	Magnetite nanoparticles	3.810	[59]
	Magnetic multiwall carbon nanotubes	16.23	[60]
	Fe_3_O_4_ nanoparticles capped with cetyltrimethylammonium bromide	18.50	[61]

## Data Availability

Date of the compounds are available from the authors.

## Sample Availability

Samples of the compounds are available from the authors.

## References

[1] Arancibia-Miranda N., Baltazar S.E., García A., Muñoz-Lira D., Sepúlveda P., Rubio M.A., Altbir D. (2016). Nanoscale zero valent supported by Zeolite and Montmorillonite: Template effect of the removal of lead ion from an aqueous solution. J. Hazard. Mater..

[2] Ma J., Zuo-Jiang S., He Y., Sun Q., Wang Y., Liu W., Sun S., Chen K. (2016). A facile, versatile approach to hydroxyl-anchored metal oxides with high Cr(VI) adsorption performance in water treatment. R. Soc. Open Sci..

[3] Mohseni-Bandpi A., Kakavandi B., Kalantary R.R., Azari A., Keramati A. (2015). Development of a novel magnetite–chitosan composite for the removal of fluoride from drinking water: Adsorption modeling and optimization. RSC Adv..

[4] Rakhunde R., Deshpande L., Juneja H.D. (2012). Chemical Speciation of Chromium in Water: A Review. Crit. Rev. Environ. Sci. Technol..

[5] Shi L.N., Zhang X., Chen Z.L. (2011). Removal ofchromium (VI) from wastewater usingbentonite-supported nanoscale zero-valent iron. Water Res..

[6] Metters J.P., Kadara R.O., Banks C.E. (2012). Electroanalytical sensing of chromium(iii) and (vi) utilising gold screen printed macro electrodes. Analyst.

[7] Zimmermann A.C., Mecabô A., Fagundes T., Rodrigues C.A. (2010). Adsorption of Cr(VI) using Fe-crosslinked chitosan complex (Ch-Fe). J. Hazard. Mater..

[8] Chen D.K., Wang H. (2015). Cr(VI) removal by combined redox reactions and adsorption using pectin-stabilized nanoscale ze-ro-valent iron for simulated chromium contaminated water. RSC Adv..

[9] Fang J., Gu Z.M., Gang D.C., Liu C.X., Ilton E., Deng B.L. (2007). Cr(VI) removal from aqueous solution by activated carbon coat-edwith quaternized poly(4-vinylpyridine). Environ. Sci. Technol..

[10] Keshmirizadeh E., Yousefi S., Rofouei M.K. (2011). An investigation on the new operational parameter effective in Cr(VI) removal efficiency: A study on electrocoagulation by alternating pulse current. J. Hazard. Mater..

[11] Wang P., Lo I.M.C. (2009). Synthesis of mesoporous magnetic gamma-Fe2O3 and its application to Cr(VI) removal from contaminated water. Water Res..

[12] Li L., Iqbal J., Zhu Y., Zhang P., Chen W., Bhatnagar A., Du Y. (2018). Chitosan/Ag-hydroxyapatite nanocomposite beads as a potential adsorbent for the efficient removal of toxic aquatic pollutants. Int. J. Biol. Macromol..

[13] Ahmed E.S., Moustafa H.Y., El-Masry A.M., Hassan S.A. (2014). Natural and synthetic polymers for water treatment against dis-solved pharmaceuticals. J. Apply. Polym. Sci..

[14] Borges O., Borchard G., Verhoef J.C., de Sousa A., Junginger H.E. (2005). Preparation of coated nanoparticles for a new mucosal vaccine delivery system. Int. J. Pharm..

[15] Khoushab F., Yamabhai M. (2010). Chitin Research Revisited. Mar. Drugs.

[16] Hacer D. (2012). Preparation and characterization of calcium alginate-based composite adsorbents for the removal of Cd, Hg, and Pb ions from aqueous solution. Toxicol. Environ. Chem..

[17] Motwani S.K., Chopra S., Talegaonkar S., Kohli K., Ahmad F.J., Khar R.K. (2008). Chitosan–sodium alginate nanoparticles as submicroscopic reservoirs for ocular delivery: Formulation, optimisation and in vitro characterisation. Eur. J. Pharm. Biopharm..

[18] Ali J., Ahmed A., Mohd S. (2020). Mesoporous Crosslinked Chitosan-Activated Charcoal Composite for the Removal of Thionine Cationic Dye: Comprehensive Adsorption and Mechanism Study. J. Polym Environ..

[19] Nasrullah A., Bhat A., Naeem A., Isa M.H., Danish M. (2018). High surface area mesoporous activated carbon-alginate beads for efficient removal of methylene blue. Int. J. Biol. Macromol..

[20] Huang C.-C., Lo S.-L., Lien H.-L. (2012). Zero-valent copper nanoparticles for effective dechlorination of dichloromethane using sodium borohydride as a reductant. Chem. Eng. J..

[21] Shu J., Cheng S., Xia H., Zhang L., Peng J., Li C., Zhang S. (2017). Copper loaded on activated carbon as an efficient adsorbent for removal of methylene blue. RSC Adv..

[22] Wang S., Vincent T., Roux J.-C., Faur C., Guibal E. (2017). Pd(II) and Pt(IV) sorption using alginate and algal-based beads. Chem. Eng. J..

[23] Vijayalakshmi K., Devi B.M., Latha S., Gomathi T., Sudha P., Venkatesan J., Anil S. (2017). Batch adsorption and desorption studies on the removal of lead (II) from aqueous solution using nanochitosan/sodium alginate/microcrystalline cellulose beads. Int. J. Biol. Macromol..

[24] Abdelmalek B.E., Sila A., Haddar A., Bougatef A., Ayadi M.A. (2017). β-Chitin and chitosan from squid gladius: Biological activities of chitosan and its application as clarifying agent for apple juice. Int. J. Biol. Macromol..

[25] Afshar H.A., Ghaee A. (2016). Preparation of aminated chitosan/alginate scaffold containing halloysite nanotubes with improved cell attachment. Carbohydr. Polym..

[26] Kavaklı C., Barsbay M., Tilki S., Güven O., Kavaklı P.A. (2016). Activation of Polyethylene/Polypropylene Nonwoven Fabric by Radiation-Induced Grafting for the Removal of Cr(VI) from Aqueous Solutions. Water Air Soil Pollut..

[27] Hassan A., Abdel-Mohsen A., Fouda M.M. (2014). Comparative study of calcium alginate, activated carbon, and their composite beads on methylene blue adsorption. Carbohydr. Polym..

[28] Badruddoza A.Z.M., Tay A.S.H., Tan P.Y., Hidajat K., Uddin M.S. (2011). Carboxymethyl-bcyclodextrin conjugated magnetic nanoparticles as nano-adsorbents for removal of copper ions: Synthesis and adsorption studies. J. Hazard. Mater..

[29] Garg V., Gupta R., Kumar R. (2004). Adsorption of chromium from aqueous solution on treated sawdust. Bioresour. Technol..

[30] Selvi K., Pattabhi S., Kadirvelu K. (2001). Removal of Cr(VI) from aqueous solution by adsorption onto activated carbon. Bioresour. Technol..

[31] Barrera-Díaz C.E., Lugo-Lugo V., Bilyeu B. (2012). A review of chemical, electrochemical and biological methods for aqueous Cr(VI) reduction. J. Hazard. Mater..

[32] Muhammad S., Alaadin A.B., Muhammad N.A. (2011). Electrocoagulation for the treatment of Wastewater for reuse in irrigation and plantation (Report). J. Basic Appl..

[33] Millar G.J., Couperthwaite S.J., Dawes L.A., Thompson S., Spencer J. (2017). Activated alumina for the removal of fuoride ions from high alkalinity groundwater: New insights from equilibrium and column studies with multicomponent solutions. Sep. Purif. Technol..

[34] Lagergren S. (1898). Zurtheorie der sogenannten adsorption gel sterstoffe. Sven. Vetensk. Handl..

[35] Ho Y., McKay G. (1999). Pseudo-second order model for sorption processes. Process. Biochem..

[36] Aljeboree M., Alshirifi N., Alkaim F. (2017). Kinetics and equilibrium study for the adsorption of textile dyes on coconut shell activated carbon. Arab. J. Chem..

[37] Elwakeel K., El-Bindary A., Kouta E., Guibal E. (2018). Functionalization of polyacrylonitrile/Na-Y-zeolite composite with amidoxime groups for the sorption of Cu(II), Cd(II) and Pb(II) metal ions. Chem. Eng. J..

[38] Ho Y.S., McKay G. (2000). The Kinetics of Sorption of Divalent Metal Ions onto Sphagnum Moss Peat. Water Res..

[39] Meenakshi S., Viswanathan N. (2007). Identification of selective ion-exchange resin for fluoride sorption. J. Colloid Interface Sci..

[40] Israa I.N., Hilal W.S. (2015). Adsorption of Eriochrom Black T Azo Dye onto Nanosized Anatase TiO2. J. Environ. Eng. Sci..

[41] Ho Y.-S. (2005). Effect of pH on lead removal from water using tree fern as the sorbent. Bioresour. Technol..

[42] Veliev E.V., Öztürk T., Veli S., Fatullayev A.G. (2006). Application of diffusion model for adsorption of azo reactrive dye on pumice. Pol. J. Environ. Stud..

[43] Daneshvar N., Salari D., Aber S. (2002). Chromium adsorption and Cr(VI) reduction to trivalent chromium in aqueous solutions by soya cake. J. Hazard. Mater..

[44] Freundlich H.M.F. (1906). Uber die adsorption in losungen. Z. Phys. Chem..

[45] Langmuir I. (1918). The adsorption of gases on plane surfaces of glass, mica and platinum. J. Am. Chem. Soc..

[46] Özcan A., Öncü E.M., Özcan A.S. (2006). Kinetics, isotherm and thermodynamic studies of adsorption of Acid Blue 193 from aqueous solutions onto natural sepiolite. Colloids Surf. A.

[47] Elgarahy A.M., Elwakeel K.Z., Elshoubaky G.A., Mohammad S.H. (2019). Microwave-accelerated sorption of cationic dyes onto green marine algal biomass. Environ. Sci. Pollut. Res..

[48] Salvestrini S., Leone V., Iovino P., Canzano S., Capasso S. (2014). Considerations about the correct evaluation of sorption ther-modynamic parameters from equilibrium isotherms. J. Chem. Thermodyn..

[49] Wentong Z., Jing Z., Wei W., Lirong M., Jianjun Z., Jimin X. (2018). Comparative study of modified/non-modified aluminum and silica aerogels for anionic dye adsorption performance. RSC Adv..

[50] Haleemat I., Folahan A., Olalekan S.F., Bhekumusa J.X. (2014). Adsorption of Cr (VI) on synthetic hematite (α-Fe2O3) nanopar-ticles of different morphologies. Korean J. Chem. Eng..

[51] Shujauddin K., Lei Z., Aimin L., Muhammad I., Xiaojuan Z. (2020). Microwave-assisted hydrothermal carbonization of furfural residue for adsorption of Cr(VI): Adsorption and kinetic study. Pol. J. Environ. Stud..

[52] Ihsanullah, Al-Khaldi F.A., Abu-Sharkh B., Abulkibash A.M., Qureshi M.I., Laoui T., Atieh M.A. (2016). Effect of acid modification on adsorption of hexavalent chromium (Cr(VI)) from aqueous solution by activated carbon and carbon nanotubes. Desalination Water Treat..

[53] Shahrina S., Lau W.J., Goha P.S., Jaafara J., Ismaila A.F. (2018). Adsorptive Removal of Cr(VI) and Cu(II) Ions from Water Solution using Graphene Oxide–Manganese Ferrite (GMF) Nanomaterials. Int. J. Eng..

[54] Pâmela B., Amanda D., Eduardo C.D., Valter A.B., Alexandre T.P. (2019). Adsorption and removal of chromium (VI) contained in aqueous solutions using a chitosan-based hydrogel. Environ. Sci. Pollut. Res..

[55] Yousef A., Hossein E., Rauf F. (2019). Enhancement removal of Cr (VI) ion using magnetically modified MgO nanoparticles. Mater. Res. Express..

[56] Malwade K., Lataye D., Mhaisalkar V., Kurwadkar S., Ramirez D. (2016). Adsorption of hexavalent chromium onto activated carbon derived from Leucaena leucocephala waste sawdust: Kinetics, equilibrium and thermodynamics. Int. J. Environ. Sci. Technol..

[57] Baghani A., Hossein M., Gholami M., Rastkari N., Delikhoon M. (2016). One-pot synthesis, characterization and adsorption studies of amine-functionalized magnetite nanoparticles for removal of Cr(VI) and Ni(II) ions from aqueousmsolution: Ki-netic, isotherm and thermodynamic studies. J. Environ. Health Sci. Eng..

[58] Padmavathy K., Madhub G., Haseena P. (2016). A study on effects of pH, adsorbent dosage, time, initial concentration and ad-sorption isotherm study for the removal of hexavalent chromium (Cr(VI)) from wastewater by magnetite nanoparticles. Procedia Technol..

[59] Huang Z.-N., Wang X.-L., Yang D.-S. (2015). Adsorption of Cr(VI) in wastewater using magnetic multi-wall carbon nanotubes. Water Sci. Eng..

[60] Elfeky S.A., Mahmoud S.E., Youssef A.F. (2017). Applications of CTAB modified magnetic nanoparticles for removal of chromium (VI) from contaminated water. J. Adv. Res..

[61] Asghar M.A., Zahir E., Shahid S.M., Khan M.N., Iqbal J., Walker G. (2018). Iron, copper and silver nanoparticles: Green synthesis using green and black tea leaves extracts and evaluation of antibacterial, antifungal and aflatoxin B1 adsorption activity. LWT.

[62] Jibran I., Noor S.S., Murtaza S., Muhammad I., Nawshad M., Fares M.H., Sara A.A., Javed A.K., Zia H.K., Amit B. (2019). Synergistic effects of activated carbon and nano-zerovalent copper on the performance of hydroxyapatite-alginate beads for the removal of As+3 from aqueous solution. J. Clean. Prod..

[63] Mohamed Aly-Eldeen A., Abeer El-Sayed A.M., Dalia M.S.A., El Zokm M.G. (2018). The uptake of Eriochrome Black T dye from aqueous solutions utilizing waste activated sludge: Adsorption process optimization using factorial design. EJABF.

